# Micro-Tomographic Investigation of Ice and Clathrate Formation and Decomposition under Thermodynamic Monitoring

**DOI:** 10.3390/ma9080668

**Published:** 2016-08-08

**Authors:** Stefan Arzbacher, Jörg Petrasch, Alexander Ostermann, Thomas Loerting

**Affiliations:** 1 Illwerke VKW Professorship for Energy Efficiency, Vorarlberg University of Applied Sciences, Hochschulstraße 1, Dornbirn 6850, Austria; stefan.arzbacher@fhv.at; 2 Institute of Physical Chemistry, University of Innsbruck, Innrain 80–82, Innsbruck 6020, Austria; 3 Department of Mathematics, University of Innsbruck, Technikerstraße 13, Innsbruck 6020, Austria; alexander.ostermann@uibk.ac.at

**Keywords:** micro-computed X-ray tomography (µCT), clathrate hydrates, ice

## Abstract

Clathrate hydrates are inclusion compounds in which guest molecules are trapped in a host lattice formed by water molecules. They are considered an interesting option for future energy supply and storage technologies. In the current paper, time lapse 3D micro computed tomographic (µCT) imaging with ice and tetrahydrofuran (THF) clathrate hydrate particles is carried out in conjunction with an accurate temperature control and pressure monitoring. µCT imaging reveals similar behavior of the ice and the THF clathrate hydrate at low temperatures while at higher temperatures (3 K below the melting point), significant differences can be observed. Strong indications for micropores are found in the ice as well as the THF clathrate hydrate. They are stable in the ice while unstable in the clathrate hydrate at temperatures slightly below the melting point. Significant transformations in surface and bulk structure can be observed within the full temperature range investigated in both the ice and the THF clathrate hydrate. Additionally, our results point towards an uptake of molecular nitrogen in the THF clathrate hydrate at ambient pressures and temperatures from 230 K to 271 K.

## 1. Introduction

Ice has been ubiquitous in colder climates worldwide and, since the advent of refrigeration, even in warmer regions [1,2]. In the universe, water is found mainly in the form of amorphous solid water (ASW) [3]. The versatility of the hydrogen bond becomes apparent when inspecting icy moons or the ice giants, which host a number of high-pressure ice polymorphs [4,5,6,7,8]. Apart from ices made of pure H_2_O, ice-like solids containing guest molecules are also seen in astrophysical environments, e.g., in cometary ice upon warming [9,10], in the mantle of icy moons [11,12,13] or on the Mars pole caps [14,15,16]. These ice-like solids are clathrate hydrates (CHs), sometimes also called gas hydrates or clathrates in short. Clathrates are inclusion compounds where a host lattice formed by water molecules provides space for guest molecules in cavities formed by tetra-, penta-, and hexagonal faces forming polyhedrons (“cages”) [17]. These do not only occur naturally in space, but also in vast amounts on Earth, in particular in the permafrost and ocean floors. Over 130 different types of guest molecules are currently known, the most prominent of them are natural gas compounds, particularly methane [18,19,20]. Although estimates of the amount of methane stored in naturally occurring clathrates vary widely, even the most conservative estimates indicate that methane clathrates are a significant natural resource [21]. Many consider the exploitation of methane clathrates in sea sediments or permafrost regions a viable route to covering the world’s energy needs in the near future [22,23,24]. Due to their ability to store hydrogen and methane at high volumetric and gravimetric energy densities, clathrates additionally constitute an interesting means for energy storage and transportation technologies [25,26,27]. Self-preservation, where some clathrates are preserved at moderate conditions, far away from their thermodynamic stability zone, further adds to their appeal [28,29,30,31,32]. The cause for self-preservation is still under investigation. A popular explanation is the formation of an ice crust that prevents further release of gas [33,34,35,36]. Promising experiments show the feasibility of an industry scale storage technology making use of this phenomenon [25,37].

In both ice and clathrate research activities, three-dimensional structural information is often obtained from diffraction measurements. Spectroscopic data derived from infrared or Raman measurements provide additional information and can help to recognize local interactions between guest and host molecules [38]. On a coarser level, micro-computed X-ray tomography (µCT) is often applied for the imaging of ice and clathrate particles. Although the resolution is far below the detailed two-dimensional information collected in cryo Scanning Electron Microscopy (cryoSEM) the additional spatial dimension and the non-destructive character of µCT makes it a relevant complementary tool. The good X-ray contrast between air and ice/snow has led to high quality results [39,40,41,42]. This non-destructive method allows for the observation of snow samples for long periods of time and has helped to learn about massive morphological changes induced in snow by temperature gradients [39,40,41,42]. Similar effects might occur in clathrate samples stored in a gaseous environment but have, to the authors knowledge, not yet been addressed in the literature.

While the X-ray contrast between clathrates and air, gas, or sediment particles is good [43,44], the contrast between clathrates and water/ice is usually poor [45,46]. Only in cases of guests with high atomic numbers (e.g., argon, krypton, and xenon) reasonable contrast is achieved [47,48,49,50]. While contrast enhancing media such as BaCl_2_ improve contrast between water-guest solutions and solid clathrate or ice particles they do not improve the contrast between ice and clathrate [51,52,53]. Diffraction-enhanced X-ray Imaging (DEI) overcomes this problem by using phase-shift information. It was widely used by Takeya et al. who achieved density resolutions of 0.01 g/cm^3^ and hence could resolve weakly absorbing clathrates and ice with a spatial resolution of 40 µm [54,55,56,57,58,59].

While not offering comparable density resolution, the setup presented here achieves significantly higher spatial resolution, which may be particularly relevant for phenomena involving a gas phase such as the temporal evolution of snow crystals or the decomposition of clathrates in air. It combines 4D-tomography (three spatial and one time dimension) as used in the µCT studies of snow metamorphism [39,40,41,42] with in situ temperature control and accurate pressure monitoring. This allows the investigation of a single clathrate sample over a long period of time in a series of µCT scans while it is subjected to tightly controlled temperature variations. At the same time, cooling power and pressure data allow the determination of phase changes and gas release. The voxel edge length of the reconstructed scans is 5 µm. This, together with the good contrast between ice/clathrate and the gaseous nitrogen, following 3D random walk segmentation [60], yields reliable volume and surface area data. Two basic types of pressure and temperature monitored experiments were carried out: (1) calorimetry-like experiments using gaseous nitrogen, water ice, THF clathrate, and 1,3-dioxolane (DXL) clathrate; and (2) tomographic observation of the decomposition of ice and THF clathrate samples, observed over a period of more than a week.

The present study demonstrates the capabilities of a novel combination of µCT and in situ thermodynamic monitoring for observing the micrometer scale transformations during slow decomposition of ice and clathrates. Since water ice and THF clathrate are widely studied and easy to handle, they are used as primary model substances. The current study concentrates on observing the evolution of the solid-gas interface. It does not aim at showing liquid–solid or solid–solid contrast. However, liquid–solid or solid–solid phase contrast may also be observed, if contrast enhancers, such as BaCl_2_ and KI [51,52,53], or high atomic number guest molecules, such as argon, krypton, and xenon [47,48,49,50], are used.

## 2. Results

### 2.1. Formation and Decay of Ice, THF and 1,3-Dioxolane Clathrate

**Figure 1:**
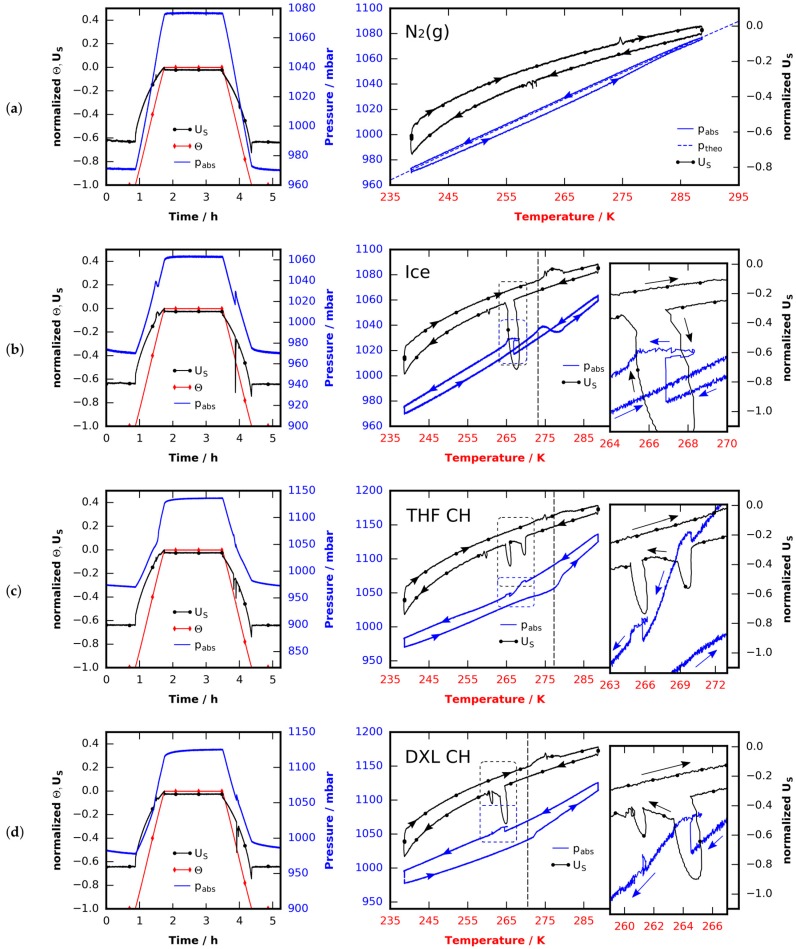
Pressure $p_{\mathrm{abs}}$, temperature Θ , and supply voltage $U_{S}$ obtained from four similar experiments done with four different samples. The samples were loaded under a dry nitrogen flow at 298 K and cooled to 238 K. The nitrogen flow ensured a dry and well defined atmosphere inside the cell. During the initial cooling phase (not shown) the cell was opened several times to compensate for thermal contraction of the gas and to ensure a constant initial pressure. After a waiting period of 30 min at 238 K the experiments were started and the data was recorded: the temperature was controlled and guided along a trapezoidal temperature profile with a bottom temperature of 238 K, a top temperature of 288 K, and a slope of 1 K/min. This was done with: (**a**) an empty cell solely filled with gaseous nitrogen; (**b**) an ice sample with mass 250 mg; (**c**) a THF clathrate sample (mole fraction 1:16.65) with mass 250 mg; and (**d**) a DXL clathrate sample (mole fraction 1:16.65) with mass 250 mg. Horizontal dashed black lines indicate the theoretical melting points of the substances used.

The performance of a custom-built pressure monitored cooling stage is assessed in four experiments using nitrogen, water-ice, THF clathrate, and 1,3-dioxolane clathrate, respectively. All experiments consist of the same three steps: (1) A sample is loaded at room temperature under a dry nitrogen flow and brought to the base temperature of 238 K; (2) A waiting period of half an hour is introduced to let the temperature field relax towards steady state before data recording is started; (3) The sample temperature is controlled to follow a trapezoidal temperature profile with base temperature 238 K, peak temperature 288 K, and a heating/cooling rate of 1 K/min (see red lines with diamond markers in Figure 1a–d). During the whole experiment the cell is tightly sealed. The assumption of a closed system is justified by the short duration of the experiments in relation to the leakage rate (see Section 4.4.2). Gas pressure inside the cell and supply voltage for thermoelectric cooling (TEC) is monitored while the sample temperature changes. Both pressure and supply voltage are shown in Figure 1a–d in two different fashions: on the left, the normalized temperature and supply voltage as a function of time; and on the right, the signals in phase space.

The first experiment (see Figure 1a) is done using dry technical nitrogen (purity ≥ 99.999%) for an estimate of the thermal gas expansion/contraction during the experiment and to test the thermal expansion model derived in Section 4.4.1. The second experiment (see Figure 1b) is used to investigate the influence of phase change. There ice is made by loading 250 ± 2.5 mg of deionized water and cooling it to the base temperature before data recording starts. Thirdly, in Figure 1c, the results of an otherwise identical experiment using 250 ± 2.5 mg of THF clathrate (mole-fraction 1:16.65) are shown. Lastly, 250 ± 2.5 mg of 1,3-dioxolane (DXL) clathrate with a mixing ration of mole fraction 1:16.65 are subjected to the same procedure (see Figure 1d). In both clathrate runs the clathrates were formed in the initial cooling phase which was not recorded.

#### 2.1.1. Thermal Expansion of Pure Nitrogen

All samples have a melting point which is well above the base temperature of 238 K. Thus, in the initial phase of the ramp the samples remain solid. The samples densities change due to thermal expansion (α ≈ 50 × 10^−6^ K^−1^ in the case of ice [61], and 30% higher in the case of THF clathrate [62,63]), however, this effect is far smaller than the thermal expansion of the nitrogen atmosphere in the cell, which dominates pressure changes at temperatures below the melting points. The temperature field inside the cell is quasi-steady but non-uniform. Therefore, a simple model based on the ideal gas law at known temperature profile is derived in Section 4.4.1. It allows the prediction of the temperature-pressure relation in the experiment. This is best observed in the case of pure nitrogen (see Figure 1a). The model pressure (dashed blue) slightly deviates from the measured pressure (solid blue) during heating due to the dynamics of the temperature field. The pressure changes and the supply voltage behavior (solid with dot marker, black) in the initial phase of the experiments are identical in all four cases (Figure 1a–d, note that different scaling factors are used). Note that during heating the supply voltage is always less negative than during cooling since heat transfer from the relatively warm surroundings through the imperfect insulation is positive.

#### 2.1.2. Melting of Ice/Clathrate

The reported melting points for ice, THF clathrate (1:17 mole fraction), and DXL clathrate (1:17 mole fraction) are 273.15 K, 277.3 K, and 270.5 K, respectively [64,65]. Once the melting points are reached, one can observe smooth melting curves, which seem to be characteristic to the substances used. In order to interpret the pressure signal one needs to consider density pairs of the solid and liquid substances at the melting point. These are (ρ_1_ = 1.000 g/cm^3^/ρ_s_ = 0.916 g/cm^3^) for ice [65], (ρ_1_ = 0.99 g/cm^3^/ρ_s_ = 0.966 g/cm^3^) for the THF clathrate, and (ρ_1_ = 0.99 g/cm^3^/ρ_s_ = 0.971 g/cm^3^) for the DXL clathrate [66]. Clathrate densities were calculated assuming a large cage occupancy factor of 16.65/17.0 in an sII-clathrate structure with unit cell volume (17.3 Å)^3^ [18]. With the substances used, melting occurs at reported melting points (within experimental error) in all cases considered. It is accompanied by an increase in density, i.e., volume contraction.

In the case of ice a slow melting can be observed at temperatures slightly above 273 K (see Figure 1b): the samples uptake of heat during melting helps the TEC to keep the sample below ambient temperature. Thus, the supply voltage is reduced (less negative) and shows a small but broad peak. Additionally, the pressure drops because liquid water has a higher density. This relates to a theoretical pressure difference of 15 mbar (cell volume 1.7 cm^3^) and is also reflected in the data. After all of the ice has become liquid, the pressure starts to rise again due to the thermal expansion of the gas.

In the case of the THF clathrate, at a temperature of 277 K, a sudden increase of pressure indicates the decomposition of the THF clathrate (see Figure 1c) since the released gaseous THF adds to the total pressure. The measured increase of 20 mbar in total pressure is much smaller than the vapor pressure of the water–THF solution (52 mbar at 277 K [67]) subtracted by the effect of volumetric contraction during melting (6 mbar). Note that during the decomposition the power supply signal shows a very small and broad peak, which is smaller than in the case of water. This reflects the difference in heat of fusion of water and THF clathrate which is 262 kJ/kg for the THF clathrate and 333.5 kJ/kg for water [64,65,68]. Furthermore, this peak in supply voltage ends at about 282 K although the pronounced increase in pressure seems to stop at a lower temperature. This indicates that a small fraction of the THF was released as gas very quickly, while the rest of it slowly dissolved in water after the melting of the clathrate.

In the case of the DXL clathrate, decomposition starts at approximately 271 K (see Figure 1d) and shows the same characteristics as the THF clathrate. The pressure increase of 10 mbar caused by a release of gaseous DXL is less pronounced than in the case of the THF clathrate. This difference can be explained by the generally lower partial pressure of DXL compared to THF. Still, the increase is lower than the vapor pressure of the DXL/water solution (17 mbar at 273 K [67]) subtracted by the result of volumetric contraction during melting (5 mbar). Note that the heat of fusion of DXL clathrate (261 kJ/kg) is almost similar to that of THF clathrate [64,68]. Also note that THF and DXL guests are capable of H-bonding to water. Such guests are known to stabilize defects in the clathrate structure which in turn leads to fast transport of guest molecules in the clathrate hydrate [38,69]. This might support the sudden initial release of guest gas in both THF and DXL clathrates, before they start to melt.

#### 2.1.3. Thermal Expansion after Melting

After melting, thermal expansion of the gaseous atmospheres and the liquid solutions are observed. Gas expansion dominates the pressure-temperature relation. With the exception of the pure nitrogen experiment at least two components constitute the gas atmosphere after melting (nitrogen–water, nitrogen–water–THF, and nitrogen–water–DXL, respectively). Although the thermal expansion model derived in Section 4.4.1 remains valid, gas composition and mass changes during heating. Thus, in order to predict the p(T) relation the change in mass and gas composition should be known. However, since in all cases more than 90% of the gas composition is formed by nitrogen and since the specific gas constant for nitrogen is more than twice those of THF and DXL (nitrogen 297 J/kg K, THF 115 J/kg K, and DXL 112 J/kg K) the change of slope of the p(T)-curve will be below 5%. This implies that the change of slope seen in the case of decomposing THF and DXL clathrate is primarily caused by an ongoing evaporation of the guest gases from the solution and not by the change in composition. Therefore, the change of slope is mainly governed by the vapor pressure $p_{v}$ of the guest/water solution. It is smallest for water ($p_{v}<\;$17 mbar for T< 288 K, see Figure 1b), medium for aqueous DXL ($p_{v}<\;$44 mbar for T< 288 K [67], see Figure 1d), and largest for aqueous THF ($p_{v}<\;$92 mbar for T< 288 K [67], see Figure 1c). An increase in pressure attributed to evaporation of gas can even be observed once the top temperature of the ramp has been reached.

The peak pressures of the individual experiments yield an estimate of the amount of gas released during the heating phase. To this end, the volumetric changes of the solid samples during melting are extrapolated from the respective melting point to the peak temperature via application of model Equation (5). It is assumed that the contraction of the sample happens immediately at the melting point and provides additional space for the nitrogen atmosphere. The volumetric contraction at the melting points has already been mentioned above. Pressures of special interest are given in Table 1.

The results for ice and THF clathrate are consistent. In both cases the full vapor pressure of the solution is not reached. This is attributed to the temperature gradient in the cell that will lead to thermodiffusion, which raises the concentration of guest molecules over the liquid/gas interface and hence reduces the vapor pressure. In contrast, in the case of the DXL clathrate the peak pressure found is slightly above the vapor pressure of the DXL water solution at 288 K.

#### 2.1.4. Thermal Contraction before Crystallization

It is assumed that when the peak temperature is reached, the guest gas concentration is saturated in all cases considered. With decreasing temperatures, the pressure reduces because of thermal contraction and a reduction of THF, DXL, and water vapor pressure. The fact that the slope of the p(T)-curve during cooling is slightly less steep than during thermal expansion after fusion may be explained by the fact that although the state changes are very slow, perfect equilibrium is not attained along the ramps.

#### 2.1.5. Crystallization

Following the temperature profile down to the base temperature, characteristic crystallization peaks can be observed. The water sample crystallizes at 266.5 K. A rapid change in density and the release of heat is seen in the inset of Figure 1b. Although the supply voltage for cooling is increased rapidly the heat cannot be removed fast enough by the TEC and hence the temperature rises. The pressure increase of 15 mbar caused by the crystallization is equal to the pressure decrease during melting. Two peaks of formation are found in the experiment with the THF clathrate (see inset of Figure 1c). The first one occurs 8 K below the melting point and can be attributed to the formation of THF clathrate. The shape of this peak is different from the peak seen in the case of pure ice. No increase in temperature can be observed. Immediately after the peak of the clathrate formation, an additional pronounced decrease of pressure indicates an ongoing but minor formation of the clathrate with THF from the atmosphere. This is followed by a small peak at approximately 265 K, showing the same characteristics as already seen with the ice peak in Figure 1b. Unlike in the case of THF clathrate the first peak of formation of DXL clathrate appears at 264 K (see inset Figure 1d). It displays the shape of an ice peak and is followed by a tiny peak at 261 K.

#### 2.1.6. Thermal Contraction after Crystallization

Reducing the temperature further again results in a contraction of the atmosphere and the solid samples. The latter is too small to be measured with the setup used. Remarkably, a hysteresis-like behavior can be seen in both the pressure as well as the supply voltage signal. The behavior of the supply voltage signal has been explained above and stems from an imperfect insulation. The hysteresis in pressure needs a more sophisticated consideration and is presumably attributed to thermal and chemical non-equilibrium.

### 2.2. Pressure Monitored µCT Imaging of Ice and THF Clathrate

**Figure 2:**
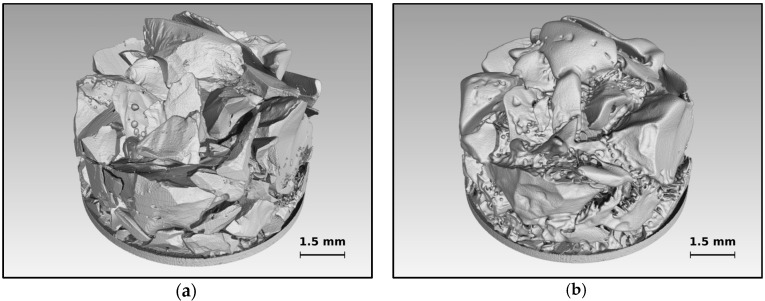
3D snapshots of ice samples obtained from µCT scans in a long-term experiment over a period of 196 h. The course of the experiment is described in the text. (**a**) Initial state of the ice after loading the sample and performing scan S1; (**b**) Final state of the ice before melting (scan S9). Although big parts of the sample sintered together the initial structure is still visible.

Ice and THF clathrate samples were investigated using a series of µCT scans over 196 h each and a prescribed temperature profile in order to investigate structural and surface changes. Simultaneously, continuous pressure monitoring allows the determination of phase changes. Figure 2 exemplarily shows results of the ice sample at two different points in time to illustrate the spatial resolution achieved.

#### 2.2.1. Ice Sample

**Figure 3:**
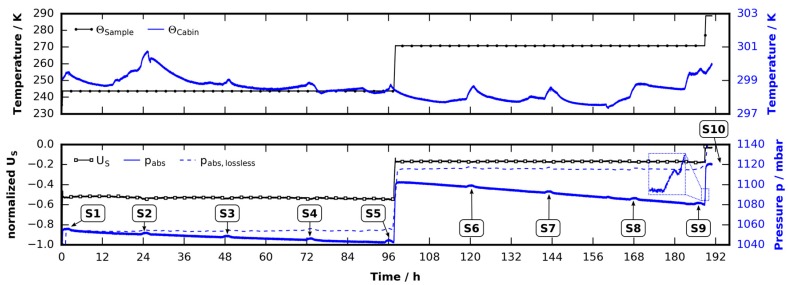
Sample and cabin temperature $\left(\Theta_{\mathrm{Sample}},\;\Theta_{\mathrm{Cabin}}\right)$ as well as cell pressure $p_{\mathrm{abs}}$ and TEC supply voltage $U_{S}$ obtained during a long-term experiment with an ice sample. The pressure has been corrected and normalized to a hypothetically constant cabin temperature (see Section 4.4.2). The markers S1–S10 indicate a series of consecutive µCT scans. The continuous decrease in pressure is due to diffusion of gas across the silicone O-ring. $p_{\mathrm{abs},\mathrm{lossless}}$ shows the pressure in a lossless cell, which is obtained by subtracting an integrated pressure loss rate ${\dot{p}}_{\mathrm{loss}}$ from $p_{\mathrm{abs}}$. Small bumps in pressure, which correspond to an increase in cell temperature are seen in each scan. This increase in temperature of approximately 0.5 K is caused by energy deposited by the X-rays. The inset at the far right shows a zoom into the pressure signal at the melting point of ice.

As a reference, ice particles are investigated. Figure 3 shows the temperature and pressure profile, corrected for pressure fluctuations caused by room temperature fluctuations (see Section 4.4.2). The ice sample was prepared as described in Section 4.1 and loaded at a temperature of 233 K together with a small amount of liquid nitrogen, which lowered the temperature of the cell to 225 K. After two minutes of waiting under a gaseous nitrogen flow the sample cell was tightly closed and heated to the start temperature of 243 K. The sample was kept at this temperature for 98 h before it was raised by 1 K/min to 270 K where it was kept for 94 h. During the overall time span of 192 h a series of nine scans, denoted by S1–S9, were carried out in 24 h intervals. After scan S9, the sample was heated above its melting point to 288 K and a last scan of the completely molten sample was conducted (S10). The melting of the ice is also visible in the pressure signal (see inset in Figure 3) which shows the same shape as already seen in the results of Section 2.1. Note the small bumps in the pressure signal during scan times, which are caused by X-ray radiation induced heating of approximately 0.5 K. Also note the rather large pressure decrease caused by the high permeability of the silicone O-ring (see Section 4.4.2). An ideal experiment with a lossless cell is modeled by the pressure signal $p_{\mathrm{abs},\mathrm{lossless}}$ in which Equation (6) is integrated over time and subtracted from $p_{\mathrm{abs}}$. The time constants used are $\alpha_{\mathrm{low}}=0.0013\;h^{-1}$ for T≤250 K and $\alpha_{\mathrm{high}}=0.0020\;h^{-1}$ for T>250 K (see Section 4.4.2). During the heating phase (after 97 h of runtime), the pressure follows the temperature ramp as predicted by the model of thermal expansion derived in Section 4.4.1. The smooth pressure signal indicates absence of phase changes up to a runtime of 190 h.

**Figure 4:**
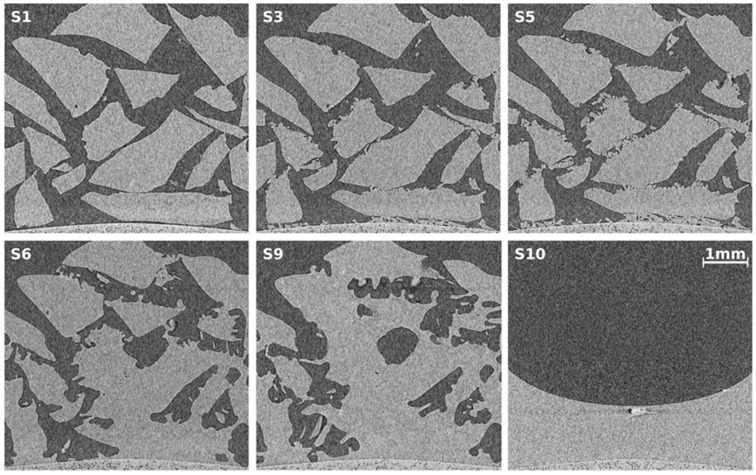
Tomographic images obtained from CT scans (numbered S1, S3, S5, S6, S9, and S10) of the ice sample at different points in time. Each slice represents the exact same position in space. In S1–S5 the sample temperature is 243 K, while in S6–S9 it is 270 K. S10 shows the meniscus of the molten sample. The bright points in slice S10 stem from tiny metallic particles that produced metal artifacts which had to be corrected manually.

Several tomographic cross-sectional reconstructions taken from selected scans are shown in Figure 4. Since the configuration of ice particles (see Figure 2) resembles a snow pack, the results are comparable with results from recent snow studies utilizing µCT [40,41,42]. Each depicted µCT slice has been taken at the identical position in space. The upper row shows significant ice crystal growth at the surfaces of the ice particles at a temperature 30 K below the melting temperature. This is similar to ref. [41], in which the temperature gradient is similar to the gradient of 0.1 K/mm found here. In this setting water vapor sublimates from tips at the surface, diffuses along the temperature gradient, and recrystallizes. Eventually this effect becomes a transport mechanism from warmer to colder ice surface sites which can also be observed in the tomograms (image bottom is the cold side). It is remarkable that under moderate conditions in snow packs this effect, also called dry snow metamorphism, results in a total replacement of 60% of the snow mass within 12 h [39].

At much higher temperatures (270 K), but still in the stability region, the ice surface becomes very mobile and tends to sinter with neighboring surfaces (see lower row, S6 and S9) which reduces surface energy [70]. At temperatures close to melting, premelted ice will exist in layers of a few nanometers in thickness [71]. Dry snow metamorphosis continues, but water vapor now evaporates from the liquid layer and recondenses elsewhere. Almost no creep is visible [72,73]. The ice particle packing keeps its configuration until it melts, which might be related to the low self-weight of the configuration.

The last slice (S10) shows the meniscus of the molten ice sample together with artifacts caused by tiny metallic particles. The artifacts are manually corrected before quantitative data analysis. Note the slightly curved bottom of the cell made of porous graphite, which is visible in all tomograms.

#### 2.2.2. THF Clathrate Sample

**Figure 5:**
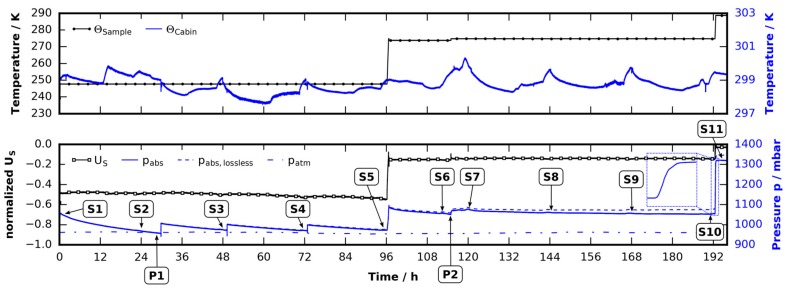
Sample and cabin temperature $\left(\Theta_{\mathrm{Sample}},\;\Theta_{\mathrm{Cabin}}\right)$ as well as cell pressure $p_{\mathrm{abs}}$ and TEC supply voltage $U_{S}$ obtained during a long-term experiment with a THF clathrate sample. The pressure $p_{\mathrm{abs}}$ has been corrected by fluctuations in cabin temperature (see Section 4.4.2). $p_{\mathrm{abs},\mathrm{lossless}}$ shows the pressure in a lossless cell, which is obtained by subtracting an integrated pressure loss rate ${\dot{p}}_{\mathrm{loss}}$ from $p_{\mathrm{abs}}$ . The markers S1–S11 indicate a series of consecutive µCT scans. Markers P1 and P2 label points of irregular pressure behavior: at P1, the cell pressure crosses the atmospheric pressure $p_{\mathrm{atm}}$, which cannot be explained by leakage. The increase in pressure at P2 is five times the increase in pressure caused by thermal expansion due to a temperature increase of 1 K. The inset at the far right shows a zoom into the pressure signal during the decomposition of the clathrate.

The experiment described in Section 2.2.1 was repeated with a THF clathrate sample. The loading procedure was identical to the ice run. Immediately after loading the sample it was heated to 247 K where it was kept for 96 hours. It was then heated to 274 K at a rate of 1 K/min. At this point the temperature was reduced immediately to 273 K again since the sample was found to be much more stable at 273 K and was stored there for 17 h. After one scan at 273 K the temperature was raised to 274 K again and four additional µCT scans of the solid clathrate were carried out within a period of 78 h. After scan S10 the temperature was raised to 288 K by 1 K/min which caused the sample to melt. A final scan (S11) was done of the liquefied sample. The temperature and pressure data of the experiment are shown in Figure 5. In comparison to the identical experiment with ice the biggest difference becomes visible in the pressure signal. The effects seen there cannot be explained by leakage alone. Almost no difference between $p_{\mathrm{abs}}$ and $p_{\mathrm{abs},\mathrm{lossless}}$ (computed using identical parameters as described in Section 2.2.1) can be observed in the first half of the experiment. This indicates an uptake of nitrogen rather than leakage and will be discussed in detail in Section 2.3.

**Figure 6:**
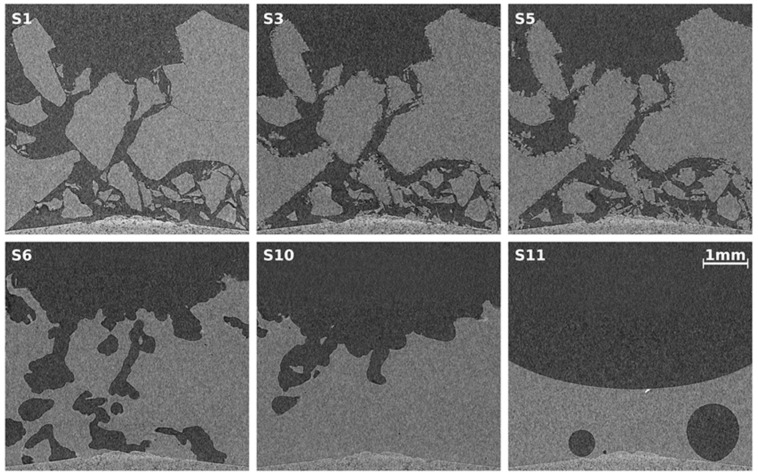
Tomographic images obtained from CT scans (numbered S1, S3, S5, S6, S10, and S11) of the THF clathrate sample at different points in time. Each slice represents the exact same position in space. In S1–S5 the sample temperature is 247 K, in S6 it is 273 K, and in S10 it is 274 K. S11 shows the meniscus of the molten sample together with gas bubbles, which can be found all over the liquefied sample. The bright point in slice S11 stems from a tiny metallic particle.

Figure 6 shows slices of reconstructed µCT scans obtained from the scans S1, S3, S5, S6, S10, and S11. The effects already seen with the ice samples recur. At low temperatures (S1–S5) one observes the growth of small crystals on top of the surface while the bulk remains unchanged. It is not possible to tell whether the crystals are ice or clathrate. However, considering their shape and growth rates they seem to be the result of the same phenomenon discussed above.

Unlike ice the THF clathrate becomes extremely mobile at a temperature 3 K below its melting point. Creep seems to be the dominant effect, which contrasts literature indicating clathrates to be more creep resistant than ice [18]. Furthermore, the clathrates self-weight is negligible. This suggests the significant settling of THF clathrate to be due to mass transport by sublimation and recondensation faster than in ice. Additionally, the approximately three orders of magnitude greater concentration of defects in the THF clathrate than in ice (a result of occasional H-bonding by THF) might also enhance the mobility of the clathrate [38,69].

Note the gas bubble in the last slice. This is remarkable, since no gas space at the bottom is visible in the slices of scan S10. Since THF is liquid at these conditions, the bubble is unlikely to be filled with gaseous THF. However, if one assumes nitrogen stored in the clathrate (either in micropores or in empty cages of the sII-structure) it could form bubbles during decomposition, especially in our case, where the sample is heated from below.

**Figure 7:**
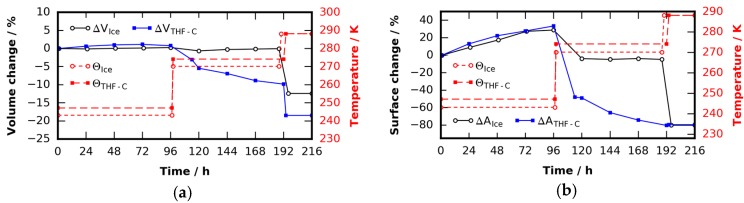
Structural analysis of the ice and THF clathrate decay study: (**a**) clathrate and ice volume changes as a function of time; and (**b**) clathrate and ice surface area changes as a function of time.

The tomographic data obtained from both experiments in Section 2.2 are analyzed further to determine volumes and surface areas. They are extracted from the scans after segmentation into gas and solid using the random walk algorithm mentioned in Section 4.5. The error of the quantitative analysis after correcting for metal particles is estimated to be less than 2%. Figure 7a shows the evolution of ice/clathrate volume, while Figure 7b shows the surface area. At temperatures 30 K below their melting point, the volume of ice/clathrate remains constant while its surface area grows. This can be related to the growth of small crystals seen in the tomograms of Figure 4 and Figure 6. Slightly below their melting points both samples begin to become mobile. In the case of ice, this mobility seems to have almost no effect on the volume. Apart from that, although significant transformations in the surface of the ice are observed, even the surface area remains stable after the temperature was changed. That is, the basic configuration of ice particles remains unchanged. In contrast, the volume and surface area of the THF clathrate decrease over time. In the last step both samples eventually become liquid. The drop in volume with the ice sample is 12% and thus 3% larger than we would expect from contraction during melting. With the THF clathrate sample a drop of 10% can be found, hence 6.5% larger than expected. We hypothesize this overestimation of volume is caused by sub micrometer-sized pores in both, the ice and the THF clathrate. These are smaller than the detection limit of the µCT setup used. While these pores are rather stable in the case of the ice, they tend to be filled over time in the case of the THF clathrate. This explains the decay in volume of the THF clathrate at a constant temperature of 274 K.

### 2.3. Nitrogen Uptake in THF Clathrate

The pressure-controlled cooling stage was tested with several different ice samples as well as THF clathrate. Whenever ice samples were investigated the pressure signal showed reproducible behavior. With THF clathrates the pressure signal of identical experiments strongly deviated from each other. These variations are attributed to the preparation process as well as the samples history. The following observations were made: (1) The longer the clathrate samples are stored in the freezer at 253 K, the higher the pressure after full decomposition of the clathrate; (2) The larger the surface area of the sample, the faster the pressure decreases in the cell; (3) The higher the cell temperature, the less pressure decrease—an unconventional reverse temperature dependence of leak rates within the range of operation; (4) Massive decreases in pressure at a temperature of 243 K do not stop at atmospheric pressure but produce negative gauge pressures far too high to be caused by thermal relaxation. Note that this can also not be explained by leakage, which actually prevents negative pressures.

**Figure 8:**
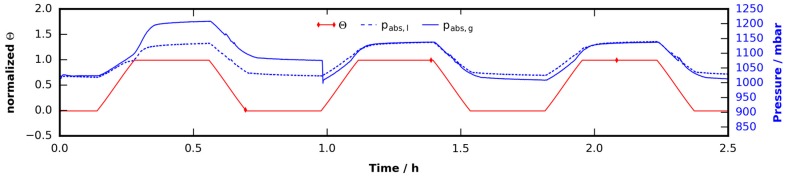
Thermal cycling of THF clathrate samples subjected to a trapezoidal temperature profile with base temperature 243 K, peak temperature 283 K, and a heating/cooling rate of 5 K/min: (1) The sample whose pressure signal has subscript “g” was stored in a freezer for one week before being loaded into the sample cell. Contact with liquid nitrogen was limited to a few seconds during the loading process. After one hour, the pressure inside the cell was relieved by quickly opening and closing it; (2) The pressure signal with subscript “l” stems from a sample which was stored in liquid nitrogen for four days after being freshly formed.

These observations can be explained if one considers an uptake of nitrogen in the THF clathrate structure. To our knowledge, this has not been observed at ambient pressure conditions in the temperature range from 230 to 273 K. However, it is known that this effect occurs at approximately 5 MPa and 268 K and has been used in a molecular sieving approach to separate gaseous hydrogen and nitrogen [74]. All observations mentioned above except for observation (2) are recorded in this study. Observation (1) is illustrated in Figure 8, which shows the pressure signal of a thermal cycling experiment with THF clathrates (cf. Section 2.1, now using a heating rate of 5 K/min, base temperature 243 K and a peak temperature of 283 K). This time, instead of freshly forming the clathrates in the sample cell, they were either stored in the freezer for one week (subscript “g” in the pressure signal) or in liquid nitrogen for four days (subscript “l” in the pressure signal), before loading them into the sample cell. In the first case contact to liquid nitrogen was limited to a few seconds during the loading process. Note the high pressure $p_{\mathrm{abs},g}$ in the first cycle after the THF clathrate decayed. The difference in pressure $p_{\mathrm{abs},g}$ from loading to the first peak is approximately 190 mbar. This is approximately 40 mbar more than the difference observed in the THF clathrate experiment of Section 2.1 where the total temperature difference was even smaller. Additionally, the pressure does not drop to its initial value but remains 40 mbar above it. This is also different from what we observed in Section 2.1 where the initial pressure was restored. After the first cycle was finished the cell was opened for one second to release the excess gas. Subsequently two additional cycles were conducted which did not show that unexpected behavior but the results known from Section 2.1. We hypothesize that the additional pressure in the first cycle was caused by nitrogen which was taken up by the clathrate during the storage time in the freezer. After decomposition, the additional nitrogen remains in the atmosphere since another uptake happens on much longer timescales than the formation of the THF clathrate. After the cell was opened, the extra nitrogen was released. The THF clathrate formed subsequently is not different from those of the experiment in Section 2.1. Remarkably, the above observations cannot be made in the case of the liquid nitrogen storage conditions, since $p_{\mathrm{abs},l}$ is entirely reversible in all three cycles.

Observations (3) and (4) are illustrated in Figure 5. The pressure decrease after the start of the experiment is much larger than in the case of the ice experiment. At the point labeled P1 the pressure eventually crosses the line of atmospheric pressure after 30 h at constant temperature. The sample cell had to be opened under a dry nitrogen flow for pressure relief since the pressure sensor used is not suitable for negative gauge pressures. This had to be repeated after 49 h and 73 h and is reflected in the kinks in the pressure signal at these times. After 96 h the sample cell was heated from 247 K to 274 K. Following the model derived in Section 4.4.1 this corresponds to an increase of 55 mbar. However, a difference in pressure of 125 mbar is observed.

At the point labeled P2, the pressure increased by 11 mbar, although the temperature was raised by only 1 K. This is five times the value predicted by the thermal expansion model. Right after scan S7, no significant changes in pressure are observed for almost 72 h. Leakage is even less than in the case of the ice sample. This can also be interpreted by a very slow release of nitrogen from the clathrate structure. During decomposition of the clathrate the pressure rises by 270 mbar while following the temperature ramp from 274 to 288 K. In the same experiment done with a freshly formed clathrate (see Section 2.1) a pressure difference of 100 mbar is seen for the same change of temperature. The pressure loss model derived in Section 4.4.2 is used to correct the pressure signal to a lossless cell. One can then sum up the remaining losses found at the temperature of 247 K and reinterpret them as a nitrogen uptake of 200 mbar. Adding these uptakes to the pressure right before the slope of the heat ramp (96 h) results in a total ramp bottom pressure of 1170 mbar. Applying the thermal expansion model to this (in two steps to include the volume contraction during melting) yields a ramp peak pressure of 1266 mbar. This is only 56 mbar below the peak pressure. This difference can be attributed to the vapor pressure of the water–THF solution and is in good agreement with the results obtained in the THF clathrate experiment of Section 2.1. Furthermore, unlike in the ice experiments, large gas bubbles are found in the molten clathrate sample (see Figure 6(S11)). No voids are found in the lower region in the last scan before melting, which could help to explain this. Thus it is assumed that the bubbles are formed during the decomposition of the clathrate by escaping nitrogen gas. Altogether, the effect is rather small: a 200 mbar uptake relates to 0.34 mL of gaseous nitrogen under standard conditions. Assuming that every empty dodecahedral cage takes up one single nitrogen molecule the total gas volume in a 250 mg sample would be 28 mL. That implies that only 1% of the empty cages are occupied, presumably in a thin layer at the surface. The depth of that layer had to be 5 µm for a surface area of 563 mm^2^ obtained from the first scan. However, in the case of micropores, the actual surface area would be much bigger and the penetration depth smaller.

We furthermore assume that this uptake did also take place in the experiments of Section 2.1 in both the THF as well as the DXL clathrate. It would explain the comparably large hysteresis in the pressure signal as well as the disproportionally high peak pressure in the DXL clathrate cycle. The latter would imply that the effect of nitrogen uptake is more pronounced in the DXL clathrate case.

## 3. Discussion

In this section, the most important results are discussed, and opportunities offered by the setup used in this study are suggested. In the first set of experiments we demonstrate that pressure and voltage signals provide critical information about the state of the sample and phase-change events. In similar experiments with differential scanning calorimeters (DSCs) or differential thermal analyzers (DTAs), one is usually unaware about pressures changes. However, in the case of forming and decomposing clathrates, an accurate pressure signal helps to understand the mechanisms involved in the formation and decomposition process. The full strength of the pressure-monitored cooling stage does not lie in the pressure signal alone. It is the provision of the complimentary quantities pressure, temperature, and power supply, which gives insight in interesting phenomena. In DSC studies of clathrate formation processes more than one peak of formation is often found [75]. It is difficult to relate, based on heat fluxes alone, these peaks to the formation of ice existing in islands, to homogeneous or heterogeneous nucleation, and to the formation of clathrates. The characteristic shapes in the pressure signal, found in our experiments, might help and can additionally provide estimates for density changes during the phase transformations.

Although it is not straightforward to interpret the pressure signal in a multicomponent gas system at non-uniform temperatures a simplified model shows good agreement with the experimental data. The application of this model helps to estimate the amount of gas evaporated in the decomposition of the clathrates. With the ice and the THF clathrate the peak pressure found is less than one would expect from the summation of thermal gas expansion and vapor pressure of the water–THF solutions. This is attributed to the effect of thermodiffusion caused by the temperature gradient in the gas volume of the cell. Conversely, in the case of DXL clathrate the peak pressure is larger than that. An explanation for this could be additional nitrogen stored in the clathrate in either pores or unoccupied cages. After decomposition this nitrogen volume would add to the total pressure. Another interesting observation is that the release of guest gas to the atmosphere does not happen immediately after melting starts. Although it is assumed that, very soon after melting starts, enough liquid is available to attain full vapor pressure, less pressure was observed in both cases.

The complementary information obtained with the cooling stage is extended by structural information gained from µCT imaging. By that it becomes possible to investigate ice and clathrate samples over a long period in a highly controlled fashion. The results show massive transformations of surface and bulk at temperatures 30 K and 3 K below the melting point. They happen on large time scales and are likely to be overlooked in short-term experiments. Results known from snow research are useful to explain not only the observations made with the ice sample, but are also applicable to the THF clathrate study. In porous media formed by snow/ice significant mass transport takes place by sublimation, temperature gradient induced diffusion, and recrystallization/recondensation. At temperatures 30 K below the melting point this process seems to be the cause of crystal growth at the ice/clathrate particles. In the case of THF clathrate particles it is yet unclear whether the crystals are formed by ice or clathrate. To our understanding, this process of metamorphism is dramatically increased with the THF clathrate at a temperature 3 K below its melting point. While the ice particle configuration is stable at the same thermal setting the configuration of THF clathrate particles collapses. Temperature gradients and higher vapor pressures at tips are the driving force for this effect. Furthermore, heat consumed or generated during sublimation and recrystallization adds to the formation of temperature gradients. To our knowledge this is not widely considered in the growth and decomposition phenomena of clathrates. Self-preservation is most prominently explained with the formation of a protecting ice layer. We propose to consider a contribution of the effect described above to the formation of such a layer since the sublimation pressure of water rises quickly from 27.7 Pa at 240 K [76].

In the attempt to explain massive deviations in the pressure signal between the ice and the THF clathrate experiments we find strong indications for an uptake of nitrogen in the THF clathrate at ambient pressures and temperatures from 230 K to 271 K. Since the total amount of nitrogen uptake is about one percent of the possible maximum we assume uptake to the surface, but no diffusion into the bulk. This correlates with the observation of increasing uptake rates with increasing surface areas. Still, the effect is rather small and quite difficult to be seen in experiments involving flow meters to determine gas release/uptake rates.

The setup presented will be useful for the investigation of many interesting phenomena. It should be straightforward to upgrade the cell with a pressure control and a valve leading to a gas analysis device. Our own plans are mainly formation and decomposition studies with different clathrates including methane, where the phenomenon of self-preservation is still not completely understood. Results from snow research suggest that temperature gradients may have tremendous influence both in formation and decomposition. These gradients are not only governed by the vicinity but by the clathrate structure itself. The method proposed in this work is perfectly suitable to study this influence. Besides that, the method could also be promising to investigate the memory effect via thoroughly designed sample cell geometries [18].

## 4. Materials and Methods

Sample preparation, the commercially available µCT and the custom built measurement cell for thermodynamic live monitoring are described.

### 4.1. Sample Preparation

Anhydrous grade tetrahydrofuran and 1,3-dioxolane, both obtained from Sigma-Aldirch (St. Louis, MO, USA), are mixed with deionized water on a Mettler-Toledo XA204DR analytical balance (Mettler-Toledo, Columbus, OH, USA). The mole fraction is 1:16.65 (THF/DXL:H_2_O) in both cases. This ensures a slight excess of guest molecules during the formation of clathrates: in both cases the stoichiometric mole fraction is 1:17. Twenty-five milliliters of each solution are stored in a refrigerator at 281 K in liquid form. The overall storage time was four weeks, it started with the experiments of Section 2.1. In those experiments the THF/DXL solutions were filled directly into the sample cell using a µL-pipette. The solid ice and clathrate particles for the experiments of Section 2.2 were obtained by freshly forming the ice/clathrates from the water/solution in a freezer at 253 K whenever needed. Chips from the frozen solution were crushed and filled into the sample cell containing some liquid nitrogen. The sample temperature was kept below 200 K in the whole filling procedure by working under liquid nitrogen.

### 4.2. Experimental Setup

#### 4.2.1. µCT Setup

A commercially available GE nanotom-m is used to obtain high-resolution micro tomographic scans of the samples [77]. Table 2 specifies the scan parameters. X-ray images from full sample rotation scans are then used to reconstruct the 3D-structure of the samples using a GPU-unit and the manufacturer’s reconstruction software datosx2 (GE Sensing & Inspection Technologies, Wunstorf, Germany).

#### 4.2.2. Pressure-Monitored Cooling Stage

**Figure 9:**
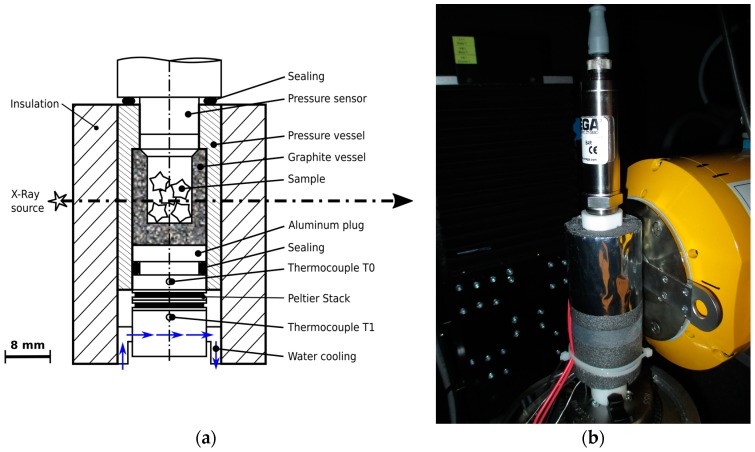
The experimental setup used in this study: (**a**) sketch of the pressure-monitored cooling stage. The dash-dotted line indicates the beam axis of the µCT; and (**b**) photograph of the setup as it is used in this work. The shiny grey part in the lower center is the radiation shield of the insulation layer of the cooling stage. The yellow part in the background is the X-ray tube of the µCT.

Figure 9 shows a sketch of the custom-made pressure-monitored cooling stage and a picture of the stage in front of the X-ray tube. The samples are placed in a graphite vessel with an inner diameter of 9 mm. Graphite is used for its unique combination of low X-ray absorption and high thermal conductivity. Since the microporous structure of graphite is not gas tight it is packed in a shell made of PEEK. The PEEK shell is connected to a pressure sensor (OMEGA PXM459-350HGI, OMEGA Engineering, Deckenpfronn, Germany) on the top side via an O-ring made of NBR. At the lower end the PEEK shell is linked to an aluminum heat sink. Here a silicone O-ring is used since the range of operation of NBR does not allow temperatures lower than −35 °C. The aluminum heat sink is cooled using a stack of two Peltier elements. Both have been purchased from Quick-Ohm, Wuppertal, Germany. The lower element (type QC-31-1.0-3.9MS) is more powerful than the upper one (type QC-17-1.4-3.7MS) since it needs to withdraw the electrical power of the upper one in addition to the heat from inside the sample cell. The current through the bottom element is set to be twice the current through the top one at all times. The upper current is set via a PID loop that controls the temperature returned from the thermocouple T0 (K-type; d=1 mm). The hot side of the Peltier stack is cooled with water, which in turn is cooled in a chiller (LAIRD MRC300, Laird, London, UK) outside of the µCT cabin. The TEC is powered by a controllable VOLTCRAFT VSP2410 laboratory power supply (Conrad Electronic AG, Wollerau, Switzerland). All sensors as well as the power pack are connected to a NI cRIO-9022 DAQ (National Instruments, Austin, TX, USA) running a LabView program to collect the data. An additional thermocouple T1 (K-type; d=1 mm) measures the temperature at the hot side of the Peltier stack. Furthermore, one thermocouple (K-type; d=1 mm) and a barometric pressure sensor (OMEGA PX419-26HBI, OMEGA Engineering, Deckenpfronn, Germany) are placed inside the µCT cabin to measure the ambient temperature and the atmospheric pressure. For clarity, “stage” always means the sum of parts illustrated in Figure 9a, while “cell” means just the volume between the base of the graphite vessel to the tip of the pressure sensor.

### 4.3. Temperature Management

**Figure 10:**
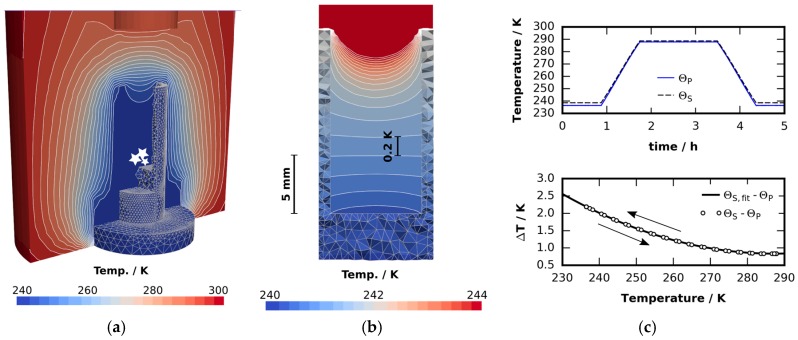
Thermal analysis of the pressure-monitored cooling stage obtained from a numerical solution of the 3D heat equation: (**a**) Temperature field inside the cooling stage as well as in the insulation layer and the tip of the pressure senor. White stars indicate the sample particles; (**b**) Temperature field inside the cell in the absence of a sample. Note the small temperature gradient of approximately 0.1 K/mm in the lower part where the particles are usually placed for the µCT investigation; (**c**) Sample temperature $\Theta_{S}$ and Peltier element temperature $\Theta_{P}$ in a thermal cycling experiment. The lower graph shows the difference $\Theta_{S}-\Theta_{P}$ as a function of $\Theta_{P}$ . No hysteresis effects can be observed.

Many of the considerations in this paper rely on an accurate knowledge of cell temperature. Almost all temperature sensors involve metals. Metals produce “metal streak artifacts” in scans where the majority of the region of interest consists of materials with little X-ray absorption, such as ice. This has to be avoided to maintain scan quality. Instead we measure the temperature below the graphite vessel containing the sample. The sample temperature is then deduced from the temperature read out of the K-type thermocouple T0. In order to do so we first determined the errors of all thermocouples using the well-defined melting points of n-decane (*T*_m_ = 243.5 K), n-dodecane (*T*_m_ = 263.6 K), and water (*T*_m_ = 273.15 K) together with the boiling point of liquid nitrogen (*T*_b_ = 77 K) [78]. A quadratic fit through these four data points was then used to relate the thermocouple read out to the actual temperature. Since the errors of the thermocouples showed a strong non-linear behavior at low temperatures, the standard deviation for the error of the quadratic fit function to the melting points is σ = 0.6 K. To compensate for dynamic effects, e.g., for the experiments described in Section 2.1, reference runs were done using the error-corrected thermocouples. The only differences between reference run and actual experiment are a surrogate pressure sensor, a different sample substance, and an additional K-type thermocouple immersed in the sample. In order to maintain a comparable thermal situation we chose a very thin thermocouple (*d* = 75 μm) for that to minimize heat flux. A glycerin–water mixture (250 mL, weight fraction 2:1) is used as a reference substance. This mixture has a freezing point of 226.7 K and lower vapor pressure than water. The specific heat of the mixture is approximately 60% of the specific heat of water [79]. The effect of varying thermal mass inside the cell can be neglected due to the small ratio of the sample and the overall thermal mass of the cooling stage. Figure 10c shows the results of this reference run. There are no differences in sample temperature during heating and cooling. The temperature of the sample is always above the temperature of the Peltier element due to the temperature gradient from the bottom to the top. This effect increases with decreasing Peltier temperature. In all experimental results discussed in this paper this difference was added to the measured temperature. This means the temperatures given in this text always relate to the sample mean temperature and are uncertain to 1 K at most.

Temperature variations in the region of interest, i.e., the graphite cup, are investigated numerically. The steady state heat conduction equation is solved using the open source 3D solver ELMER [80]. Convection inside the cell can be neglected due to stable temperature stratification and the small Grasshof number of the problem. A temperature boundary condition T=240 K at the cold side of the Peltier stack as well as a heat transfer boundary condition to the ambient (ambient temperature$\;T_{a}=298$ K, heat transfer coefficient (*h* = 25 W/m^2^K) is applied. Figure 10a,b shows the temperature field inside the cell from simulation. The simulated temperature variation in the lower third of the cell is less than 1 K.

### 4.4. Pressure Management

The pressure sensor is used to detect the pressure effects of phase transitions and gas release. The pressure signal is also influenced by the temperature dependent behavior of the dry nitrogen taking up the rest of the cell. In addition, the low temperatures at the bottom side of the cell require using a high permeability silicone O-ring. This causes considerable leakage. Hence we require: (1) a model for thermal expansion of the nitrogen gas; and (2) quantitative information of leakage during the experiments.

#### 4.4.1. Thermal Expansion and Contraction in Non-Uniform Temperature Fields

When applying a state equation, e.g., the ideal gas law, to the measurement cell, the non-uniform temperature field must be accounted for.

The temperature field T(x) illustrated in Figure 10 is considered. Since the gas inside the cell is motionless, the pressure must be constant throughout the cell. Hydrostatic pressure variations are neglected. Locally, the ideal gas law states the pressure

(1) p(x)=p=ZρRT(x),

where ρ is the gas density, R is the mass specific gas constant, and Z is the compressibility factor of a non-ideal gas. Assuming an absolutely tight cell, mass conservation and integration over the cell volume V yields

(2) $$m=\underset{V}{\int }\rho \;dV=\underset{V}{\int }\frac{p}{ZRT}\;dV=\frac{p}{ZR}\underset{V}{\int }\frac{1}{T\left(x\right)}\;dV$$

for the total gas mass m. Reformulation of Equation (2) leads to

(3) $$p=Z\;m\;R\cdot {\left(\underset{V}{\int }\frac{1}{T\left(x\right)}\;dV\right)}^{-1}.$$

The pressure can then be calculated for any known inhomogeneous temperature field given mass m either by numerical or analytical integration. The temperature field in Figure 10b is approximated by a one-dimensional temperature field, neglecting radial gradients

(4) $$T\left(z\right)=\;\{\begin{array}{c} T_{0}\;\;\;\;\mathrm{for}\;\;0<z\leq h_{0},\;\;\;\\ \;\;\;\;\;T_{0}+\frac{z-h_{0}}{h_{1}-h_{0}}\cdot \left(T_{1}-T_{0}\right)\;\;\;\;\mathrm{for}\;h_{0}<z\leq h_{1}. \end{array}$$

It considers two regions: (1) the region inside the graphite cup ($0<z\leq h_{0}$) where a uniform temperature field is assumed; and (2) the region from $h_{0}\;$to the tip of the pressure sensor with a linear temperature variation. Integration yields

(5) $$p=\;Z\;m\;R\cdot {\left(\frac{A_{0}h_{0}}{T_{0}}+A_{1}\left(h_{1}-h_{0}\right)\cdot \frac{\ln T_{1}-\ln T_{0}}{T_{1}-T_{0}}\right)}^{-1},$$

where $A_{0}$ and $A_{1}$ refer to the cross sectional areas of the two regions. Equation (5) is used as a model for thermal expansion and contraction in this study. $A_{0},\;A_{1},\;h_{1},\;$and the total volume of the cell are determined using the tomographic reconstruction of the empty cell. The value $h_{0}=3$ mm was extracted from a fit to the data obtained in the empty cell experiment of Section 2.1 and used in every other occasion although with loaded samples some of the lower volume is occupied by the sample instead of nitrogen.

#### 4.4.2. Pressure Loss due to Leakage

**Figure 11:**
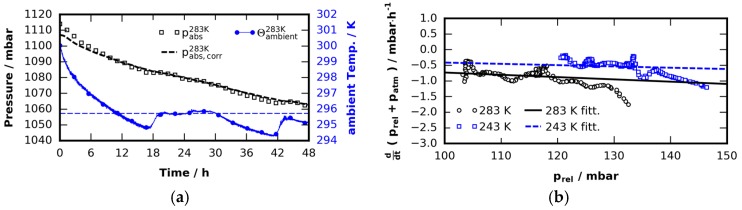
Long-term pressure decay analysis: (**a**) the influence of temperature fluctuations of the ambient temperature (blue line with dot markers) becomes visible in the pressure signal (square markers). A correction (discussed in the text) of this effect is possible (dashed black line); and (**b**) the sum of gauge and atmospheric pressure change rate, obtained from pressure tests using an empty cell at different temperatures are shown. The linear fit functions to the data directly yield the loss rate.

In order to consider pressure losses, pressure fluctuations introduced into the system by changes in ambient temperature have to be subtracted. Equation (5) can be used to extract the current total gas mass m, which changes over time due to leakage, evaporation and condensation. This mass is then again inserted in Equation (5), where $T_{0}$ is still the bottom temperature but $T_{1}$ is a fixed mean ambient temperature. By that the pressure has been corrected to a constant ambient temperature $T_{1}$. A result of this procedure is depicted in Figure 11a. After the pressure signal is corrected by fluctuations in ambient temperature, the linear ansatz

(6) $${\dot{p}}_{\mathrm{loss}}=-\alpha \;p_{\mathrm{rel}}$$

is used to model pressure loss rate as a function of gauge pressure $p_{\mathrm{rel}}$. The rate of change of $p_{\mathrm{rel}}$ is

(7) $${\dot{p}}_{\mathrm{rel}}={\dot{p}}_{\mathrm{loss}}-{\dot{p}}_{\mathrm{atm}},$$

it depends on leakage as well as atmospheric pressure $p_{\mathrm{atm}}$. Inserting Equation (6) in Equation (7) yields

(8) $${\dot{p}}_{\mathrm{rel}}+{\dot{p}}_{\mathrm{atm}}=-\alpha \;p_{\mathrm{rel}}.$$

Both gauge pressure and atmospheric pressures are measured during the experiments and thus also their rates of change are known. The sum of ${\dot{p}}_{\mathrm{rel}}$ and$\;{\dot{p}}_{\mathrm{atm}}$ are obtained from a series of independent pressure experiments using an empty cell at two different temperatures. Numerical differentiation of the pressure signal was done after filtering the data with a Savitzky–Golay filter and a subsequent cubic spline interpolation [81]. The measured loss rates strongly depend on the age of the O-rings. While new O-rings show almost no loss, rings that were in heavy use show significant loss. The worst results are displayed in Figure 11b as a function of $p_{\mathrm{rel}}$ and provide an upper bound on the amount of leakage. A linear fit to that data yields the time constants $\alpha_{243}=0.00412\pm 0.00001\;h^{-1}$ (cell bottom temperature 243 K) as well as $\alpha_{283}=0.00731\pm 0.00001\;h^{-1}$ (cell bottom temperature 283 K). From this we get the pressure loss rate as a function of the gauge pressure. Note that the loss rate at higher temperatures is slightly larger than at lower temperatures. This was found in all pressure tests and is probably related to the generally higher gas permeability of silicone at higher temperatures [82]. Nevertheless, since the sealing force will also be reduced at lower temperatures this effect will be small if present at all [83].

### 4.5. Image Reconstruction and Post Processing

**Figure 12:**
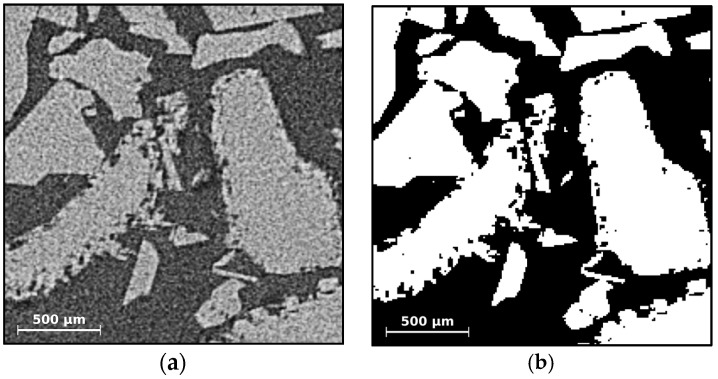
Segmentation of noisy CT images using the random walk segmentation filter described by Grady [52]: (**a**) Original CT slice taken from scan S5 of the THF clathrate experiment of Section 2.2.2. The bright phase is the THF clathrate, the dark phase is gaseous nitrogen; (**b**) The same slice after segmentation.

Image stacks obtained from µCT are reconstructed using GE’s phoenix datosx2 reconstruction software (GE Sensing & Inspection Technologies, Wunstorf, Germany). In a first step a smaller region of interest, containing solely the interior of the graphite sample cell, is extracted from the 3D raw data. A Gaussian filter with the smallest possible kernel size of three voxels is then applied to reduce the noise level while maintaining all details. Image stacks (8-bit, jpg) are then written and analyzed in an in-house post processing toolbox. Random walk segmentation as presented by Grady [52] is applied in 3D situations which requires efficient memory management and solution methods for very large linear systems with approximately 300 × 10^6^ degrees of freedom. Figure 12 shows the result of the random walk segmentation on a single CT slice obtained from a THF clathrate sample of the experiment described in Section 2.2.2.

Sample volumes are calculated based on the segmented data by voxel counting. The surface area *A* is computed using the derivative of the 2-point probability function, S_2_, at its origin [84,85]:

(9) $$A=-4\cdot {\frac{dS_{2}}{dr}|}_{r=0}.$$

The 2-point probability function is computed for distances of 0 and 0.9 voxel side lengths using Monte Carlo integration with 10 million sampling points each. The derivative is approximated by the difference quotient of these two points.

## Figures and Tables

**Table 1:** Pressure values as extracted from Figure 1a–d.

Pressure (mbar)	Ice	THF CH	DXL CH
at ramp bottom	969	969	977
at ramp top	1064	1137	1126
at melting point (solid samples)	1041	1049	1045
extrapolated from melting point (liquid samples) to ramp top	1052	1063	1072
left for guest gas	12	74	54
vapor pressure over solution at ramp top	17	92	44

**Table 2:** CT settings used in all experiments.

Parameter	Value
tube voltage (kV)	70
focal spot size (µm)	2.5
magnification factor (-)	20
voxel edge length (µm)	5.0
no. of images per scan (-)	1500
exposure time per image (ms)	1000
no. of averaged images per increment (-)	3
